# Silent scars: understanding interpersonal sensitivity, paranoid ideation, and hostility from adverse childhood experiences in Jamaica

**DOI:** 10.3389/fpsyg.2025.1547926

**Published:** 2025-09-17

**Authors:** Rosemarie Johnson, Elroy Galbraith, Roger Carl Gibson, Tracey-Ann Coley

**Affiliations:** ^1^Department of Sociology, Psychology & Social Work, Faculty of Social Sciences, The University of the West Indies, Kingston, Jamaica; ^2^Aeon Technology Solutions Limited, Kingston, Jamaica; ^3^Department of Community Health and Psychiatry, The University of the West Indies, Kingston, Jamaica

**Keywords:** adverse childhood experiences, interpersonal sensitivity, hostility, paranoid ideation, violence, Jamaica

## Abstract

**Background:**

Adverse childhood experiences (ACEs) are known to predict adverse outcomes related to physical and mental health, including anxiety and depression. How ACEs predict the outcomes of interpersonal sensitivity, paranoid ideation, and hostility, which are known to be associated with impaired interpersonal relationships, interpersonal conflict and violence, is less well researched. Consequently, this study aimed to determine the extent to which the number and types of ACEs that individuals experienced were associated with these outcomes, and whether these relationships were moderated by the sociodemographic variables of age, sex, educational level, and relationship status.

**Method:**

The study used data from a survey of 1,633 adult Jamaicans who constituted a non-probability sample. They were recruited via flyers that were placed on social media, at doctor's offices, in supermarkets, at places of work, and at educational institutions. The survey consisted of sociodemographic items, as well as the Adverse Childhood Experiences International Questionnaire (ACE-IQ) and the Symptom Checklist-90-Revised (SCL-90-R). A correlational design, using Pearson's correlation analyses, was used to assess the association between overall past ACEs and specific current interpersonal psychopathological vulnerabilities, namely interpersonal sensitivity, paranoid ideation, and hostility. Regression analyses were also used to determine which specific childhood adversities were associated with these vulnerabilities.

**Results:**

Most participants (70.5%) reported having experienced at least four of the 13 categories of ACEs explored in the ACE-IQ. There were positive correlations among the ACE-IQ and SCL-90-R subscales of interest (interpersonal sensitivity, paranoid ideation, and hostility), with correlation coefficients ranging from 0.249 to 0.770 (*p* < 0.001). Emotional abuse was the most commonly reported ACE (70.5%), followed by violence in the home (69.3%), and community violence (66.9%). Seven of the 13 ACEs from the ACE-IQ were associated with all three mental health outcomes. Physical abuse had an inverse relationship with paranoid ideation, as did household alcohol or drug misuse with interpersonal sensitivity and hostility. The relationships between ACEs and the interpersonal psychopathological vulnerabilities were not moderated by the sociodemographic variables.

**Conclusions:**

The number of ACEs (ACE-IQ score) was positively correlated with all three psychopathological outcomes. Many ACEs were associated with one or more of these outcomes. A few ACES exhibited an inverse relationship with either paranoid ideation or hostility and interpersonal sensitivity. These findings add new knowledge to an under-explored area and are discussed in relation to prior research, theory, and practice.

## Introduction

The association between childhood trauma and poor health outcomes in later life is well known, and the systematic consideration of multiple Adverse Childhood Experiences (ACEs) has guided comprehensive explorations of these issues in various settings and contexts (Hughes et al., 2017). Some adverse mental health outcomes of ACEs that have been widely researched include depression, anxiety, post-traumatic stress, and substance misuse (Chang et al., 2019; Leza et al., 2021; Abate et al., 2025). Less well researched are interpersonal psychopathological vulnerabilities such as interpersonal sensitivity, paranoid ideation, and hostility.

Trauma Theory posits that traumatic experiences, such as those events that are considered adverse childhood experiences (ACEs), can impede the psychological wellbeing of individuals through the development of hyperarousal, constriction, and intrusion (Bloom, 2019; Basham, 2022). Trauma-induced hyperarousal produces a prolonged state of self-protective vigilance that may be difficult to terminate or regulate. In response to this hyperarousal, individuals may also experience constriction, wherein they become physiologically, emotionally, and cognitively unresponsive to stimuli. While this functionally protective maneuver may help individuals avoid painful trauma-related responses temporarily, intrusions and psychological disruptions may still surface and overwhelm them. These disruptions often challenge their fundamental beliefs about safety and trust, stripping them of control, connection, and meaning in life. As a result, individuals who have experienced childhood adversity may be highly sensitive to possible threats of danger and feel as if danger is always present. They may also adopt a defensive or hostile stance to protect themselves against these perceived threats (Bloom, 2019; Basham, 2022). Consequently, themes of interpersonal sensitivity, paranoid ideation, and hostility may be prevalent in the experiences and interactions of persons who have been affected by childhood adversity.

In the context of this study, interpersonal sensitivity is characterized by feelings of inadequacy and inferiority resulting in self-doubt, self-consciousness, and negative expectations about others' behavior and perceptions of them. Paranoid ideation refers to a disordered mode of thinking that is marked by suspiciousness, projective thinking, and a fear of loss of autonomy that is not necessarily of delusional intensity. On the other hand, hostility is manifested as thoughts, feelings or actions that are driven by irritability, rage, resentment, or similar emotions.

Gilbert et al. (2006) found that individuals with higher levels of interpersonal sensitivity had the propensity to over-interpret, and attach an inappropriately high level of significance to, the words, behaviors, and responses of others. Such individuals are also more vulnerable to the harmful effects of disapproval, rejection, and interpersonal conflict, which in turn can cause them to exhibit aggressive behaviors. Nickerson et al. (2013) report that in the aftermath of trauma, interpersonal sensitivity heightens the risk of aggressive retaliation. Research also suggests that paranoid ideation may have a significant adverse impact on individuals' relationships and interactions with others, as well as heighten their risk of demonstrating hostility and violence toward others (Chine et al., 2016).

Other research findings have demonstrated strong associations between ACEs or other traumatic experiences and the interpersonal psychological vulnerabilities of interpersonal sensitivity, paranoid ideation, and hostility. Johns et al. (2019) found that among female inmates who had been exposed to intimate partner violence, ACEs were correlated to both interpersonal sensitivity and hostility. Post-traumatic stress disorder has also been linked to interpersonal sensitivity by Slanbekova et al. (2019). In addition, hostility has been demonstrated as having an association with ACEs in young adults (Lin and Chiao, 2024), while victimization by bullying in childhood has shown some association with both interpersonal sensitivity and paranoid ideation (McDonnell et al., 2018).

There have been very few studies that have focused on the simultaneous examination of interpersonal sensitivity, paranoid ideation, and hostility together with multiple ACEs. However, one notable study, conducted by Prachason et al. (2024), found that physical neglect in males and emotional abuse in females were correlated with interpersonal sensitivity, paranoid ideation and hostility, as well as other psychopathological dimensions, among a Belgian twin cohort. Further exploration of interpersonal sensitivity, paranoid ideation, and hostility and their possible association with multiple ACEs would be valuable, especially in unexplored socio- cultural contexts like Jamaica. As noted by James-Myers et al. (2021), psychological outcomes are the interaction of many factors that relate to individual differences, social, cultural, spiritual, and political factors. Therefore, examining ACEs using samples from various geographic regions and biographical subgroups can lead to broader perspectives on the impact of trauma.

Research on interpersonal sensitivity, paranoid ideation, hostility, and ACEs is particularly relevant to Jamaica given that a significant proportion of the population has experienced adversity during childhood (Smith and Mosby, 2003; Fray et al., 2022; Lee et al., 2022) and that there is limited research on the prevalence of psychopathologies among Jamaican survivors of childhood adversity. Of note, research in this area has focused on certain ACE categories like physical, sexual, and emotional abuse and their associations with anxiety, depression, and substance abuse (Lee et al., 2022; Longman-Mills et al., 2015), leaving unanswered the question of the cumulative impact of ACEs on other aspects of psychological functioning. Furthermore, given the high prevalence of violence in Jamaica (Campbell and Harriott, 2024) and the significant potential impact of interpersonal sensitivity, paranoid ideation, and hostility on interpersonal relationships and aggression, an exploration of possible associations between ACEs and these psychopathological traits in this population would be valuable.

The relationship between ACEs and psychopathological outcomes may be influenced by demographic factors. Whitaker et al. (2021) found synergistic effects between female sex and ACEs when they were examined as risk factors for depression and anxiety. In addition, Davidson and Hall (1995) found that males are more prone to exhibit outward expressions of hostility, such as aggression or anger, while females tend to internalize their feelings. Females are also more prone to heightened sensitivity to interpersonal cues, such as criticism or rejection (Davidson and Hall, 1995). Regarding the influence of age, Gooding et al. (2012) found that older adults used a wider array of effective coping strategies than younger adults. This suggests that older adults would have fewer psychological sequelae from stress and adversity. Another demographic factor that might influence the relationship between ACEs and psychopathological outcomes is educational attainment. Specifically, educational achievement can be viewed as an indicator of functionality due to the goal-directed behaviors and resiliency necessary for success. Given that psychopathological states are generally associated with dysfunction, it is possible that educational level could moderate the relationship between ACEs and psychopathological dimensions. Lastly, having an intimate partner may allow for emotional, instrumental, and social support (Holt-Lunstad et al., 2008; Ta-Johnson et al., 2017), thus mitigating the association between trauma and the development of psychopathological traits.

In this study, we aimed to determine the prevalence of ACEs experienced by the sample and the extent to which the number and types of ACEs were associated with levels of interpersonal sensitivity, paranoid ideation, and hostility. More specifically the research questions were:

1. What were the number and types of ACEs that participants experienced?
2. What levels of paranoid ideation, interpersonal sensitivity, and hostility did participants have?
3. Was there a relationship between the number of ACEs and paranoid ideation, interpersonal sensitivity, and hostility?
4. To what extent were specific ACEs associated with levels of paranoid ideation, interpersonal sensitivity, and hostility?
5. Did age, sex, educational level, and relationship status moderate the relationship between ACEs and paranoid ideation, interpersonal sensitivity, and hostility?

## Materials and methods

### Participants

One thousand, six hundred and thirty-three Jamaicans took part in this study. They were predominantly females (64.3%) ranging in age from 18 to 81 years, with a mean age of 27 (SD = 11.9) years. Most respondents completed college or had a graduate education (76.6%). Most were single (63.9%), followed by those who were either married or in common-law relationships (13.0%).

### Measures

#### Adverse childhood experiences international questionnaire (ACE-IQ)

The World Health Organization (WHO) developed the ACE-IQ for adults 18 years of age and older (World Health Organization, 2018). It is a self-report inventory that evaluates 29 types of early childhood adversity which it subsumes under 13 categories. The number of types of adversity that constitute each category ranges from one to four, depending on the category. The 13 categories are: physical abuse, emotional abuse, contact sexual abuse, household alcohol or drug misuse, having an incarcerated household member, chronic mental illness in the household, violence in the home, parental separation/divorce/death, emotional neglect, physical neglect, bullying, exposure to community violence, and exposure to collective violence. Each of the 29 types of adversity is probed by a single item, with either binary response options (yes/no; five items) or frequency response options that range from “many times” to “never” (24 items). The WHO's binary scoring approach was used in this study. In this approach, responses in the affirmative to any of the items included in a category resulted in a score of one for that category (indicating that exposure to the category was present). Otherwise, the category received a score of zero (indicating that exposure to the category was absent). The resulting ACE-IQ summary score is the sum of the scores for each category and ranges from 0 to 13. The score also represents the number of ACE categories that were experienced. [Cronbach's alpha or other traditional measures of internal consistency were not applied to the ACE-IQ because they would not have been appropriate, given the nature of the data and the method of scoring].

#### Symptom checklist-90-revised (SCL-90-R)

The SCL-90-R is a 90-item self-report symptom inventory designed to reflect the psychological symptom patterns of community, medical, and psychiatric respondents (Derogatis, 1994). It has nine primary symptom dimensions: Somatization, Obsessive-Compulsive, Interpersonal Sensitivity, Depression, Anxiety, Hostility, Phobic Anxiety, Paranoid Ideation, and Psychoticism. For this study, participants' responses were only examined for the symptom dimensions of interpersonal sensitivity—nine items, paranoid ideation—six items, and hostility—six items. The SCL-90-R utilizes a five-point distress scale from 0, meaning “not at all,” to 4, meaning “extremely.” Means are computed for each dimension and converted to a Standard T-Score with a mean of 50 and a Standard Deviation of 10. A T-Score of 63 and above is considered to reflect a risk of the psychopathological dimension measured by the scale. For each of the psychopathological dimensions, higher scores are indicative of greater levels of symptom severity. Internal consistency for the SCL-90-R subscales used in this study was good to excellent: interpersonal sensitivity [Cronbach's α = 0.90, 95% CI (0.89, 0.90)], hostility [α = 0.84, 95% CI (0.83, 0.85)], and paranoid ideation [α = 0.80, 95% CI (0.79, 0.82)].

### Procedures

Participants completed an anonymous survey consisting of sociodemographic items, the ACE-IQ, and the SCL-90-R over 2 years, from 2019 to 2021. After ethical approval was obtained from the researchers' university (ECP 218, 16/17), participants were recruited to participate in the study. Flyers were made and distributed at college and university lecture halls, doctors' offices, supermarkets, human resource departments of companies, and church bulletin boards. Notices were also placed on the intranet, and social media platforms of the researchers' university (Facebook, LinkedIn, Instagram), as well as the social media platforms of one of the researchers.

The flyer for the research directed interested participants to a Survey Monkey link to the survey questionnaire, as well as to the contact information of the principal investigator. Research assistants followed up with persons who had contacted the principal investigator to facilitate their completion of the survey in person. This was a non-probability method of sampling, which yielded a sample that was not representative of the national population, but that, nevertheless, facilitated the pursuit of the research objectives. Sixty percent of the questionnaires were completed on Survey Monkey. Financial support for data collection was provided through a grant from the University of the West Indies, Mona.

### Data analysis

Prior to analysis, the raw dataset underwent a series of data cleaning and preparation steps to ensure data quality and integrity. Firstly, participants under the age of 18 years were excluded, unless age was missing. Categorical variables were recoded for consistency and meaningful grouping: education was categorized into “College/University,” “Post-graduate,” and “Less than university;” and relationship status into “Married/Common-law,” “Divorced/Separated,” and “Single.” Age was grouped into four categories: “18–9,” “30–4,” “45–9,” “60–1.” A missing value analysis revealed no systematic patterns, suggesting that missingness was likely “missing completely at random” (MCAR). For statistical testing, missing numerical values were imputed using the median, while descriptive statistics retained the original missing values. Additionally, diagnostic tests were performed to check for violations of assumptions for the statistical tests.

Data analysis was structured in multiple phases. Initially, descriptive statistics were computed to summarize the demographic characteristics of the participants, the distribution of responses for the ACE-IQ, and the distribution of scores on the SCL-90-R dimensions of interest (interpersonal sensitivity, paranoid ideation, and hostility). Pearson correlation analyses were conducted to evaluate the relationships among the ACE-IQ summary scores and the SCL-90-R dimensions of interest, with the significance level set at alpha = 0.05.

To examine the relationship between ACEs and adult psychopathologies (measured by the SCL-90-R), multivariable linear regression models were employed using all 13 ACE-IQ categories as independent variables and the SCL-90-R dimensions as dependent variables. All missing values were imputed using the median for the variable. Also, multicollinearity among the independent variables was examined using variance inflation factors (VIFs). Thresholds of VIF < 5 were adopted as indicators of an absence of problematic multicollinearity.

An a priori power analysis was conducted using G^*^Power 3.1 (Faul et al., 2007) for a multiple regression model (fixed model, R^2^ deviation from zero) with 13 independent variables. Assuming a medium effect size (*f*^2^ = 0.15), α = 0.05, and desired power of 0.95, the analysis indicated that a minimum of 189 participants would be needed. Our sample of 1,633 participants greatly exceeded this threshold, providing robust statistical power for detecting medium-sized effects.

To specifically investigate the potential moderating roles of socio-demographic variables (age, sex, educational level, and relationship status) on the association between ACEs and psychological outcomes, moderation analyses were conducted using additional multivariable linear regression models. This approach, consistent with standard moderation analysis methodology, involved creating interaction terms between each socio-demographic variable and the ACE-IQ scores. These interaction terms, along with the main effects of both the ACE-IQ scores and the respective socio-demographic variable, were included as independent variables in the regression models with SCL-90-R scores as dependent variables. A statistically significant interaction term would indicate that the relationship between childhood adversities and adult psychological functioning was indeed moderated by the corresponding socio-demographic characteristic. This method aligns with established practices for testing moderation using multiple regression, as outlined by Baron and Kenny (1986) and Aiken and West (1991). An alpha level of 0.05 was utilized to determine statistical significance for all analyses.

## Results

The participants experienced all categories of adversities, and some categories were more prevalent than others. For example, emotional abuse was the most commonly reported category of adversity (70.5%). Violence in the home (69.3%), community violence (66.0%), physical abuse (65.8%), and bullying (56.6%) were also experienced by most of the participants. Most participants (70.5%) also reported adverse experiences in four or more ACE-IQ categories, and a few (18.6%) reported no ACE exposures.

The levels of risk of interpersonal sensitivity, hostility, and paranoid ideation, as determined by the established SCL-R-90 T-score threshold of 63 varied among the outcomes assessed. It was 30.9% for interpersonal sensitivity, 26.3% for paranoid ideation, and 22.7 % for hostility.

ACE-IQ summary scores correlated positively with interpersonal sensitivity, *r* = 0.638, *p* < 0.001, *r*^2^ = 0.407, hostility, *r* = 0.587, *p* < 0.001, *r*^2^ = 0.345, and paranoid ideation, *r* = 0.249, *p* < 0.001, *r*^2^ = 0.062. Interpersonal sensitivity, hostility, and paranoid ideation also correlated positively with each other with statistically significant correlation coefficients ranging from 0.549 to 0.770 (Table 1).

**Table 1:** Correlates and 95% CI among ACE-IQ summary scores, interpersonal sensitivity, hostility, and paranoid ideation.

**Variable**	**1**	**2**	**3**	**4**
ACE-IQ summary score	–	0.638^**^ [0.608–0.666]	0.587^**^ [0.554–0.618]	0.249^**^ [0.203–0.294]
Interpersonal sensitivity		–	0.770 ^**^ [0.750–0.789]	0.604^**^ [0.572–0.634]
Hostility			–	0.549 ^**^ [0.514–0.582]
Paranoid ideation				–

^**^p < 0.001, *n* = 1,633. 1 = ACE-IQ summary score, 2 = Interpersonal sensitivity, 3 = Hostility, 4 = Paranoid ideation.

Table 2 presents the results of the multivariable regression analyses. For interpersonal sensitivity, the overall model explained a significant proportion of its variance, *R*^2^ = 0.455, *p* < 0.001, indicating that approximately 45.5% of the variance in interpersonal sensitivity can be attributed to the included ACE domains. Among the individual independent variables, bullying emerged as having the most robust association (*B* = 7.045, *p* < 0.001). This positive association indicated that individuals exposed to victimization by bullying tend to report heightened interpersonal sensitivity. Similarly, emotional abuse (*B* = 4.962, *p* < 0.001), violence in the home (*B* = 4.149, *p* < 0.001), and collective violence (*B* = 3.588, *p* < 0.001) also showed significant positive associations, indicating that individuals exposed to these adversities tend to report heightened interpersonal sensitivity.

**Table 2:** Multivariable regression analyses with ACEs as independent variables and each SCL-90-R dimension as the dependent variable.

**Variable**	**Interpersonal sensitivity B [95% CI]**	**Hostility B [95% CI]**	**Paranoid ideation B [95% CI]**
Physical abuse	0.97 [−0.51, 2.45]	1.35 [−0.08, 2.78]	−2.41^**^ [−3.87, −0.96]
Emotional abuse	4.96^**^ [3.12, 6.81]	3.06^**^ [1.27, 4.84]	0.11 [−1.70, 1.92]
Contact sexual abuse	2.38^**^ [1.19, 3.58]	2.70^*^ [1.55, 3.86]	1.88^*^ [0.71, 3.05]
Household alcohol/drug misuse	−2.41^*^ [−4.15, −0.66]	−2.61^*^ [−4.30, −0.93]	−0.88 [−2.59, 0.84]
Incarcerated household member	1.06^*^ [0.29, 1.83]	1.09^*^ [0.34, 1.84]	1.19^*^ [0.43, 1.95]
Chronic mental illness in household	1.06^*^ [0.29, 1.83]	1.09^*^ [0.34, 1.84]	1.19^*^ [0.43, 1.95]
Violence in home	4.15^**^ [2.45, 5.84]	3.77^**^ [2.13, 5.41]	2.45^*^ [0.78, 4.11]
Parental divorce/separation/death	0.50 [−0.62, 1.63]	0.12 [−0.97, 1.20]	−1.02 [−2.12, 0.08]
Emotional neglect	3.12^**^ [1.52, 4.71]	0.92 [−0.63, 2.46]	2.76^**^ [1.19, 4.33]
Physical neglect	2.39^**^ [0.84, 3.93]	3.04^**^ [1.54, 4.53]	3.91^**^ [2.39, 5.42]
Bullying	7.05^**^ [5.89, 8.20]	4.76^**^ [3.64, 5.88]	2.80^**^ [1.67, 3.94]
Community violence	2.21^**^ [0.89, 3.53]	2.87^**^ [1.60, 4.15]	−0.75 [−2.04, 0.54]
Collective violence	3.59^**^ [2.33, 4.85]	3.41^**^ [2.18, 4.63]	3.04^**^ [1.80, 4.27]
*R^2^* (*N*)	0.455^**^ (1,633)	0.380^**^ (951)	0.115^**^ (759)

^*^p < 0.01; ^**^p < 0.001.

Other independent variables with significant positive associations with interpersonal sensitivity included contact sexual abuse (*B* = 2.382, *p* < 0.001), emotional neglect (*B* = 3.116, *p* < 0.001), physical neglect (*B* = 2.388, *p* = 0.002), and community violence *(B* = 2.211, *p* = 0.001). Notably, having an incarcerated household member (*B* = 1.06, *p* = 0.007) and chronic mental illness in the household (*B* = 1.06, *p* = 0.007) also showed significant positive associations, albeit with smaller effect sizes. Conversely, the presence of alcohol or drug misuse in the household was negatively associated with interpersonal sensitivity (*B* = −2.407, *p* = 0.007), suggesting that individuals who experienced these forms of adversity reported lower levels of interpersonal sensitivity. Other ACEs, including physical abuse (*p* = 0.198) and parental divorce/separation/death (*p* = 0.380), were not significantly associated with interpersonal sensitivity, indicating that their effects may be less direct or are accounted for by other ACEs in the model.

For hostility, the overall regression model explained a significant proportion of its variance, *R*^2^ = 0.380, *p* < 0.001, indicating that approximately 38.0% of the variance in hostility can be attributed to the included ACE domains. Among the individual independent variables, bullying emerged as having the most robust association (*B* = 4.757, *p* < 0.001). This positive association indicated that individuals exposed to victimization by bullying tend to report heightened hostility. Violence in the home (*B* = 3.766, *p* < 0.001), collective violence (*B* = 3.405, *p* < 0.001), and emotional abuse (*B* = 3.057, *p* < 0.001) also showed significant positive associations with hostility.

Other independent variables with significant positive associations with hostility included physical neglect (*B* = 3.037, *p* < 0.001), community violence (*B* = 2.871, *p* < 0.001), and contact sexual abuse (*B* = 2.701, *p* = 0.002). Having an incarcerated household member (*B* = 1.09, *p* = 0.004) and chronic mental illness in the household (*B* = 1.09, *p* = 0.004) also showed significant positive associations, albeit with smaller effect sizes. Conversely, the presence of alcohol or drug misuse in the household was negatively associated with hostility (*B* = −2.614, *p* = 0.001), suggesting that individuals who experienced this form of adversity reported lower levels of hostility. Other ACEs, including physical abuse (*p* = 0.065) parental divorce/separation/death (*p* = 0.831), and emotional neglect (*p* = 0.245) were not significantly associated with hostility, suggesting that their effects may be less direct or are accounted for by other ACEs in the model.

Regarding paranoid ideation, the overall regression model explained 11.5% of its variance (*R*^2^ = 0.115, *p* < 0.001). Among the individual independent variables, physical neglect emerged as having the most robust association (*B* = 3.908, *p* < 0.001). This positive association indicated that individuals exposed to physical neglect tend to report heightened paranoid ideation. Similarly, collective violence (*B* = 3.036, *p* < 0.001), bullying (*B* = 2.804, *p* < 0.001), emotional neglect (*B* = 2.757, *p* < 0.001), and violence in the home (*B* = 2.446, *p* < 0.004) also showed significant positive associations with paranoid ideation.

Other independent variables with significant positive associations with paranoid ideation included chronic mental illness in the household (*B* = 1.188, *p* < 0.002), incarcerated household member (*B* = 1.188, *p* = 0.002), and contact sexual abuse (*B* = 1.881, *p* = 0.002). Conversely, physical abuse was negatively associated with paranoid ideation (*B* = −2.414, *p* = 0.001), suggesting that individuals who experienced this form of adversity reported lower levels of paranoid ideation. Other ACEs, including emotional abuse (*p* = 0.903), parental divorce/separation/death (*p* = 0.070), alcohol or drug misuse in the home (*p* = 0.315), and community violence (*p* = 0.255) were not significantly associated with paranoid ideation, suggesting that their effects may be less direct or are accounted for by other ACEs in the model.

Collinearity diagnostics indicated that most independent variables had VIF values close to 1, with all below 5. Two variables (incarcerated household member and chronic mental illness in the household) returned undefined VIFs due to redundancy in coding, but inspection confirmed that this did not affect estimation. Multicollinearity among the independent variables was, therefore, not a concern in the models.

A summary of the moderation analyses that were conducted to examine whether the relationship between ACEs and the SCL-R-90 dimensions was moderated by key demographic variables (sex, age, educational attainment, and relationship status) is shown in Table 3. A statistically significant interaction term in these models would provide evidence that the demographic variables moderated the relationship between ACEs and the psychopathological outcomes. Across all models, ACEs emerged as being consistently associated with elevated psychological distress, while demographic moderators showed limited evidence of interaction effects.

**Table 3:** Summary of moderation analyses: ACEs and their association with psychological outcomes.

**Outcome variable**	**ACEs main effect (p)**	**Significant moderators (main effects)**	**Significant interactions (moderation)**	***R^2^***
Interpersonal sensitivity	*p* < 0.001	Post-graduate Education (↓, *p* = 0.026)	None	0.147–0.211
Hostility	*p* < 0.001	Age 30–44 and 45–59 (↓, *p* < 0.05)	None	0.121–0.173
Paranoid ideation	*p* < 0.001	Age 30–44 (↓, *p* < 0.001); Post-grad Edu (↓, *p* = 0.034)	Age 30–44 × ACEs (*p* = 0.030)	0.140–0.186

Only significant results are shown, please see the Supplementary material for the full results.

Regarding interpersonal sensitivity, exposure to ACEs was significantly associated with higher levels of reported sensitivity across all models. The inclusion of gender, age, education, and relationship status as moderators revealed minimal differential effects. Specifically, neither gender nor relationship status significantly moderated the association between ACEs and interpersonal sensitivity (*p* > 0.05). While post-graduate education was independently associated with lower interpersonal sensitivity (*B* = −9.95, *p* = 0.026), the interaction between ACEs and educational attainment was non-significant, suggesting that higher education does not substantially buffer the psychological effects of early adversity. Age group also did not significantly moderate the ACE–interpersonal sensitivity relationship.

Similar patterns were observed in the models regarding hostility. ACEs were significantly associated with higher hostility scores (*p* < 0.001) across all models, confirming the detrimental impact of childhood adversity on affect regulation. However, the interaction terms between ACEs and gender, age, education, and relationship status were all non-significant (*p* > 0.05), suggesting that these demographic variables do not meaningfully alter the strength of this relationship. Although younger participants (ages 30–44 and 45–59) exhibited significantly lower hostility scores compared to the reference group, these effects did not extend to interaction terms, indicating no differential effect of ACEs across age groups.

In the models regarding paranoid ideation, ACEs again emerged as being strongly associated (*p* < 0.001). Most demographic moderators did not significantly interact with ACEs, with the exception of age. A significant interaction was observed between ACEs and the 30–44 age group (*B* = 0.87, *p* = 0.030), indicating a slightly stronger association between ACEs and paranoid ideation in this cohort. However, this effect was not observed in older age groups. Educational attainment and relationship status, while sometimes associated with main effects, did not significantly moderate the association between ACES and paranoid ideation.

Taken together, these results indicate that the psychological impact of ACEs on interpersonal sensitivity, hostility, and paranoid ideation is robust and largely invariant across sociodemographic subgroups. The consistent and significant main effects of ACEs across all outcomes underscore their pervasive influence on adult psychological functioning. While some demographic variables such as education and age were associated with differences in outcome levels, they did not meaningfully alter the strength of the relationship between ACEs and psychological distress.

## Discussion

This study examined the under-explored outcomes of interpersonal sensitivity, paranoid ideation, and hostility in relation to exposure to ACEs among a sample of Jamaican adults. The results indicated that most participants (81.4%) reported exposure to at least one ACE and 70.5% of them had exposure to at least four ACEs. The prevalence of four or more ACES was both lower and higher than rates reported in other studies. Whereas, the rate from the current study is high in comparison to national studies, e.g., Swedo et al. (2023) who reported a US rate of 17%, it is lower than the rate (92.4%) from a study with a non-probability sample from Mexico (Sánchez-Jáuregui et al., 2023). In another study of participants from a chronic-disease cohort in Chile (Santelices et al., 2025), there were five ACE-IQ categories which were reported as being prevalent by more than half of the sample; the same number of categories with a prevalence of 50% or more also applies to the current study.

The most frequently occurring ACE-IQ category for participants from the current study was emotional abuse which involves being yelled and screamed at, as well as being insulted and humiliated. Other frequently occurring experiences were violence in the home, physical abuse, and bullying. These findings are also consistent with previous research in various settings (Fray et al., 2022; Sánchez-Jáuregui et al., 2023; Santelices et al., 2025). The high prevalence of emotional abuse can be interpreted within the broader psychosocial dynamics of Caribbean child-rearing practices. For example, according to Hickling and Hutchinson (2012), many traditional Caribbean parenting styles are influenced by post-colonial legacies and authoritarian social norms. Parenting is therefore often characterized by physical discipline, verbal humiliation, and emotional distancing which are used in an attempt to instill obedience and resilience in children. However, these methods, which are often perceived as culturally normative, may unintentionally perpetuate a cycle of emotional and verbal abuse.

The proportion of respondents who were above the risk threshold (T > 63) for interpersonal sensitivity, paranoid ideation, and hostility varied. The psychological outcome that was associated with the highest percentage of participants above the risk threshold was interpersonal sensitivity (30.9%). Although this proportion is relatively low, it suggests that many of the respondents had intense feelings of inadequacy and inferiority, self-doubt, self-deprecation, and marked discomfort during interpersonal interactions. Another perspective would be to focus on the many respondents (69.1%) who did not have high levels of interpersonal sensitivity. These two contrasting perspectives may be reconciled if individual variability in reactions to adversity are considered, as well as the cultural context within which the adversity occurs. Indeed, some persons may use adversity as psychological fuel to overcome challenges (Consoli and Myers, 2021). The findings also highlight the fact that an individual's response to adversity is influenced by many factors and that traumatic experiences do not automatically lead to sustained psychological challenges. Similar arguments could be brought to bear on the findings of low levels of risk for paranoid ideation (26.3%) and hostility (22.7%).

The low prevalence of persons above the risk thresholds for the interpersonal psychopathological vulnerabilities should also be considered within the context of Trauma Theory which suggests that traumatic experiences like ACEs may cause individuals to create protective maneuvers to help avoid painful trauma-related responses. For this sample, further exploration beyond a self-report questionnaire would also add to the understanding of the questions of coping, adaptation and resilience.

The strong correlation among scores of interpersonal sensitivity, paranoid ideation, and hostility is in keeping with past research. It has previously been noted that interpersonal sensitivity and hostility are behavioral traits that do not exist in isolation (Anli and Sar, 2017) but frequently interact and influence each other in various ways (Bonab and Koohsar, 2011; Davidson and Hall, 1995). The correlation among the interpersonal psychopathological vulnerabilities also suggests that there is a high level of comorbidity among affected persons. Clinicians may be guided by this finding in their approach to patients or clients with these issues.

As anticipated, ACE-IQ scores had significant positive correlations with the interpersonal psychopathological vulnerabilities that were measured with the SCL-90-R. In addition, the regression analyses showed seven of the 13 ACE-IQ categories to be associated with all three SCL-90-R dimensions that were explored. These ACE categories were contact sexual abuse, incarceration of a member of the household, chronic mental illness in the household, violence in the home, physical neglect, bullying, and collective violence. These findings are all in keeping with the Trauma Theory model which posits that trauma induces hyperarousal together with disruptions in beliefs about safety and trust that generate heightened sensitivity, paranoid thinking and hostility (Bloom, 2019; Basham, 2022). These findings also align with other emerging research that has directly explored associations between childhood adversity and these types of psychopathological outcomes (Prachason et al., 2024). As previously noted, paranoid ideation, interpersonal sensitivity, and hostility are likely to have an adverse impact on interpersonal relationships. The extent to which ACEs result in impaired interpersonal relationships; the roles of hostility, interpersonal sensitivity, and paranoid ideation in the association; and the factors that might mitigate these relationships are interesting areas for further study.

Another noteworthy finding is the negative correlation between physical abuse and self-reports of paranoid ideation. This suggests that participants who experienced physical abuse (which the ACE-IQ defines as being spanked, slapped, kicked, punched, beaten, or hit or cut with an object) had lower levels of paranoid ideation (under which the SCL-90-R subsumes projective thoughts, disordered thinking, suspiciousness, fear of loss of autonomy, and delusions). Many of the physically abusive behaviors mentioned above are commonly used as disciplinary measures for children in Jamaican households (Smith and Mosby, 2003), and this may be one of the reasons that the ACE-IQ identified physical abuse in many of the respondents. One possible explanation for the negative correlation observed in this sample is that individuals who endured physically abusive behaviors may have cultivated resilience mechanisms that enabled them to cope with stress and adversity, potentially leading to a reduction in paranoid ideation. Examining the presence of support systems, coping strategies, or positive influences in their lives could yield valuable insights. Another consideration is the nature of self-reports which can sometimes lead to underreporting or misreporting of paranoid symptoms due to stigma, lack of insight, or emotional regulation strategies. In summary, while this finding might initially seem counterintuitive, it reveals the complexity of human psychological responses and indicates that further in-depth studies are necessary to explore the underlying mechanisms and nuanced interpretations of the impact of childhood trauma on paranoid ideation. It also underscores the importance of clinicians considering individual, social, and cultural factors that are involved in the presentation of psychological challenges.

Additional negative correlations were found between alcohol or drug misuse in the household and both interpersonal sensitivity and hostility. These findings suggest that a higher level of exposure to household alcohol or drug misuse was associated with lower levels of interpersonal sensitivity and hostility, and further reflect the complexity of human psychological responses. While these findings may seem counterintuitive, they could have resulted from the psychological blunting maneuver that is sometimes seen when individuals experience intense trauma, as explained by Trauma Theory.

The absence of moderation effects of age, sex, educational attainment, and relationship status on the association between ACEs and the interpersonal psychopathological vulnerabilities may be related to the profound and enduring effect of ACEs on psychological outcomes, as reported in previous research (Nurius et al., 2012). Another consideration is that Jamaican society is marked by strong communal ties that are generally supportive and cut across age, sex, and educational lines (Forsythe-Brown et al., 2017). The existence of these social networks may equalize stress-coping across demographic groups thereby eliminating the demographic moderation of the effects of ACEs.

Some limitations apply to the current study. Persons with significant childhood adversities may have been more inclined to participate in this study, and this may have artificially inflated the prevalence of ACEs. Also, participants in the survey were not representative of the Jamaican population, e.g., most of them were women (64.3%) and were highly educated with college and post-graduate degrees (76.6%), thus limiting the generalizability of the results. Another consideration is that retrospective self-reports may introduce recall and response bias. The information that is requested on the ACEs questionnaire is also very sensitive and that could have influenced the way participants responded, especially those who completed the survey in person. Because persons with internet access and related devices could access the survey more easily than those relying on the in-person method, it is possible that this biased the sample; persons of higher socioeconomic status have greater access to relevant resources and may have been over-represented.

Limitations notwithstanding, the findings add to the evidence of linkages between ACEs and interpersonal sensitivity, paranoid ideation and hostility, and point to potentially helpful strategies for diminishing interpersonal violence. Policies and lifestyle practices aimed at addressing, preventing, and mitigating the harmful effects of ACEs and the negative sequalae of mental and behavioral outcomes are a matter of urgency. Additionally, public education on the impact of ACEs, recognizing risk factors in self and others, and providing the appropriate psychosocial supports is important to address this public health issue.

## Data Availability

The raw data supporting the conclusions of this article will be made available by the authors, without undue reservation.
