# Supplementary material for: Silent scars: understanding interpersonal sensitivity, paranoid ideation, and hostility from adverse childhood experiences in Jamaica
Source: Front Psychol. 2025 Sep 17;16:1547926. doi: 10.3389/fpsyg.2025.1547926 (PMC12484013; doi:10.3389/fpsyg.2025.1547926)
Supplement: Supplementary file 1 [file Table_1.docx]

# Full Results of Moderation Analyses

OLS Regression Results

===============================================================================

Dep. Variable: T_INTERSONALSCALE_2 R-squared: 0.147

Model: OLS Adj. R-squared: 0.145

Method: Least Squares F-statistic: 76.32

Date: Tue, 18 Mar 2025 Prob (F-statistic): 1.51e-45

Time: 22:49:50 Log-Likelihood: -5112.3

No. Observations: 1333 AIC: 1.023e+04

Df Residuals: 1329 BIC: 1.025e+04

Df Model: 3

Covariance Type: nonrobust

================================================================================================

coef std err t P>|t| [0.025 0.975]

------------------------------------------------------------------------------------------------

Intercept 47.8312 0.956 50.028 0.000 45.956 49.707

q0002[T.Male] -1.7423 2.179 -0.799 0.424 -6.018 2.533

ACEIQBINARY_v2 2.0077 0.149 13.438 0.000 1.715 2.301

ACEIQBINARY_v2:q0002[T.Male] 0.0723 0.343 0.211 0.833 -0.601 0.746

==============================================================================

Omnibus: 17.927 Durbin-Watson: 1.671

Prob(Omnibus): 0.000 Jarque-Bera (JB): 12.437

Skew: -0.110 Prob(JB): 0.00199

Kurtosis: 2.581 Cond. No. 48.5

==============================================================================

Notes:

[1] Standard Errors assume that the covariance matrix of the errors is correctly specified.

OLS Regression Results

===============================================================================

Dep. Variable: T_INTERSONALSCALE_2 R-squared: 0.211

Model: OLS Adj. R-squared: 0.206

Method: Least Squares F-statistic: 48.67

Date: Tue, 18 Mar 2025 Prob (F-statistic): 1.71e-61

Time: 22:49:50 Log-Likelihood: -4869.5

No. Observations: 1284 AIC: 9755.

Df Residuals: 1276 BIC: 9796.

Df Model: 7

Covariance Type: nonrobust

=====================================================================================================

coef std err t P>|t| [0.025 0.975]

-----------------------------------------------------------------------------------------------------

Intercept 49.4226 0.990 49.915 0.000 47.480 51.365

age_group[T.30-44] -8.7152 2.991 -2.914 0.004 -14.583 -2.847

age_group[T.45-59] -6.2456 2.597 -2.405 0.016 -11.340 -1.151

age_group[T.60-81] -8.8231 4.455 -1.981 0.048 -17.562 -0.084

ACEIQBINARY_v2 1.9844 0.156 12.699 0.000 1.678 2.291

ACEIQBINARY_v2:age_group[T.30-44] 0.4275 0.416 1.029 0.304 -0.388 1.243

ACEIQBINARY_v2:age_group[T.45-59] -0.4276 0.429 -0.996 0.320 -1.270 0.415

ACEIQBINARY_v2:age_group[T.60-81] 0.0430 0.848 0.051 0.960 -1.621 1.707

==============================================================================

Omnibus: 9.747 Durbin-Watson: 1.821

Prob(Omnibus): 0.008 Jarque-Bera (JB): 7.596

Skew: -0.090 Prob(JB): 0.0224

Kurtosis: 2.669 Cond. No. 99.1

==============================================================================

Notes:

[1] Standard Errors assume that the covariance matrix of the errors is correctly specified.

OLS Regression Results

===============================================================================

Dep. Variable: T_INTERSONALSCALE_2 R-squared: 0.181

Model: OLS Adj. R-squared: 0.178

Method: Least Squares F-statistic: 58.56

Date: Tue, 18 Mar 2025 Prob (F-statistic): 3.64e-55

Time: 22:49:50 Log-Likelihood: -5072.6

No. Observations: 1330 AIC: 1.016e+04

Df Residuals: 1324 BIC: 1.019e+04

Df Model: 5

Covariance Type: nonrobust

===============================================================================================================

coef std err t P>|t| [0.025 0.975]

---------------------------------------------------------------------------------------------------------------

Intercept 54.3733 4.232 12.847 0.000 46.070 62.676

educ[T.Post-graduate degree] -9.9484 4.463 -2.229 0.026 -18.704 -1.193

educ[T.University] -5.4898 4.372 -1.256 0.209 -14.067 3.088

ACEIQBINARY_v2 1.1671 0.558 2.092 0.037 0.073 2.262

ACEIQBINARY_v2:educ[T.Post-graduate degree] 0.7829 0.602 1.300 0.194 -0.398 1.964

ACEIQBINARY_v2:educ[T.University] 0.8885 0.584 1.521 0.129 -0.258 2.035

==============================================================================

Omnibus: 14.091 Durbin-Watson: 1.783

Prob(Omnibus): 0.001 Jarque-Bera (JB): 9.757

Skew: -0.073 Prob(JB): 0.00761

Kurtosis: 2.607 Cond. No. 196.

==============================================================================

Notes:

[1] Standard Errors assume that the covariance matrix of the errors is correctly specified.

OLS Regression Results

===============================================================================

Dep. Variable: T_INTERSONALSCALE_2 R-squared: 0.201

Model: OLS Adj. R-squared: 0.198

Method: Least Squares F-statistic: 65.39

Date: Tue, 18 Mar 2025 Prob (F-statistic): 5.34e-61

Time: 22:49:50 Log-Likelihood: -4959.0

No. Observations: 1305 AIC: 9930.

Df Residuals: 1299 BIC: 9961.

Df Model: 5

Covariance Type: nonrobust

=====================================================================================================================

coef std err t P>|t| [0.025 0.975]

---------------------------------------------------------------------------------------------------------------------

Intercept 48.9907 5.970 8.206 0.000 37.279 60.703

relationship[T.Married/Common-law] -8.2592 6.280 -1.315 0.189 -20.579 4.060

relationship[T.Single] 0.0263 6.044 0.004 0.997 -11.830 11.882

ACEIQBINARY_v2 1.1595 0.798 1.453 0.147 -0.406 2.725

ACEIQBINARY_v2:relationship[T.Married/Common-law] 0.9530 0.854 1.116 0.265 -0.723 2.629

ACEIQBINARY_v2:relationship[T.Single] 0.8602 0.812 1.060 0.290 -0.732 2.453

==============================================================================

Omnibus: 17.218 Durbin-Watson: 1.748

Prob(Omnibus): 0.000 Jarque-Bera (JB): 11.802

Skew: -0.101 Prob(JB): 0.00274

Kurtosis: 2.580 Cond. No. 294.

==============================================================================

Notes:

[1] Standard Errors assume that the covariance matrix of the errors is correctly specified.

OLS Regression Results

==============================================================================

Dep. Variable: T_HOSTILITYSCALE_2 R-squared: 0.121

Model: OLS Adj. R-squared: 0.119

Method: Least Squares F-statistic: 61.26

Date: Tue, 18 Mar 2025 Prob (F-statistic): 4.28e-37

Time: 22:49:50 Log-Likelihood: -5053.4

No. Observations: 1333 AIC: 1.011e+04

Df Residuals: 1329 BIC: 1.014e+04

Df Model: 3

Covariance Type: nonrobust

================================================================================================

coef std err t P>|t| [0.025 0.975]

------------------------------------------------------------------------------------------------

Intercept 47.7802 0.915 52.232 0.000 45.986 49.575

q0002[T.Male] -3.1067 2.085 -1.490 0.136 -7.197 0.984

ACEIQBINARY_v2 1.6634 0.143 11.636 0.000 1.383 1.944

ACEIQBINARY_v2:q0002[T.Male] 0.0757 0.329 0.230 0.818 -0.569 0.720

==============================================================================

Omnibus: 29.734 Durbin-Watson: 1.890

Prob(Omnibus): 0.000 Jarque-Bera (JB): 16.284

Skew: 0.055 Prob(JB): 0.000291

Kurtosis: 2.470 Cond. No. 48.5

==============================================================================

Notes:

[1] Standard Errors assume that the covariance matrix of the errors is correctly specified.

OLS Regression Results

==============================================================================

Dep. Variable: T_HOSTILITYSCALE_2 R-squared: 0.173

Model: OLS Adj. R-squared: 0.169

Method: Least Squares F-statistic: 38.24

Date: Tue, 18 Mar 2025 Prob (F-statistic): 6.81e-49

Time: 22:49:50 Log-Likelihood: -4817.2

No. Observations: 1284 AIC: 9650.

Df Residuals: 1276 BIC: 9692.

Df Model: 7

Covariance Type: nonrobust

=====================================================================================================

coef std err t P>|t| [0.025 0.975]

-----------------------------------------------------------------------------------------------------

Intercept 49.1492 0.951 51.702 0.000 47.284 51.014

age_group[T.30-44] -7.3938 2.872 -2.575 0.010 -13.027 -1.760

age_group[T.45-59] -5.2256 2.493 -2.096 0.036 -10.117 -0.334

age_group[T.60-81] -7.7805 4.277 -1.819 0.069 -16.171 0.610

ACEIQBINARY_v2 1.6069 0.150 10.710 0.000 1.313 1.901

ACEIQBINARY_v2:age_group[T.30-44] 0.4035 0.399 1.011 0.312 -0.379 1.186

ACEIQBINARY_v2:age_group[T.45-59] -0.5487 0.412 -1.331 0.184 -1.357 0.260

ACEIQBINARY_v2:age_group[T.60-81] -0.1446 0.814 -0.178 0.859 -1.742 1.453

==============================================================================

Omnibus: 23.040 Durbin-Watson: 2.038

Prob(Omnibus): 0.000 Jarque-Bera (JB): 13.133

Skew: -0.011 Prob(JB): 0.00141

Kurtosis: 2.505 Cond. No. 99.1

==============================================================================

Notes:

[1] Standard Errors assume that the covariance matrix of the errors is correctly specified.

OLS Regression Results

==============================================================================

Dep. Variable: T_HOSTILITYSCALE_2 R-squared: 0.147

Model: OLS Adj. R-squared: 0.144

Method: Least Squares F-statistic: 45.68

Date: Tue, 18 Mar 2025 Prob (F-statistic): 1.29e-43

Time: 22:49:50 Log-Likelihood: -5018.6

No. Observations: 1330 AIC: 1.005e+04

Df Residuals: 1324 BIC: 1.008e+04

Df Model: 5

Covariance Type: nonrobust

===============================================================================================================

coef std err t P>|t| [0.025 0.975]

---------------------------------------------------------------------------------------------------------------

Intercept 48.9910 4.064 12.054 0.000 41.018 56.964

educ[T.Post-graduate degree] -2.5995 4.285 -0.607 0.544 -11.007 5.808

educ[T.University] -1.5716 4.199 -0.374 0.708 -9.808 6.665

ACEIQBINARY_v2 1.4851 0.536 2.772 0.006 0.434 2.536

ACEIQBINARY_v2:educ[T.Post-graduate degree] -0.2127 0.578 -0.368 0.713 -1.347 0.922

ACEIQBINARY_v2:educ[T.University] 0.3811 0.561 0.679 0.497 -0.719 1.482

==============================================================================

Omnibus: 24.555 Durbin-Watson: 1.944

Prob(Omnibus): 0.000 Jarque-Bera (JB): 14.314

Skew: 0.059 Prob(JB): 0.000779

Kurtosis: 2.506 Cond. No. 196.

==============================================================================

Notes:

[1] Standard Errors assume that the covariance matrix of the errors is correctly specified.

OLS Regression Results

==============================================================================

Dep. Variable: T_HOSTILITYSCALE_2 R-squared: 0.139

Model: OLS Adj. R-squared: 0.136

Method: Least Squares F-statistic: 42.02

Date: Tue, 18 Mar 2025 Prob (F-statistic): 3.39e-40

Time: 22:49:50 Log-Likelihood: -4933.9

No. Observations: 1305 AIC: 9880.

Df Residuals: 1299 BIC: 9911.

Df Model: 5

Covariance Type: nonrobust

=====================================================================================================================

coef std err t P>|t| [0.025 0.975]

---------------------------------------------------------------------------------------------------------------------

Intercept 47.4015 5.856 8.094 0.000 35.913 58.890

relationship[T.Married/Common-law] -4.2709 6.160 -0.693 0.488 -16.356 7.814

relationship[T.Single] 0.7146 5.928 0.121 0.904 -10.915 12.345

ACEIQBINARY_v2 1.1083 0.783 1.416 0.157 -0.428 2.644

ACEIQBINARY_v2:relationship[T.Married/Common-law] 0.5839 0.838 0.697 0.486 -1.060 2.228

ACEIQBINARY_v2:relationship[T.Single] 0.5874 0.796 0.738 0.461 -0.975 2.150

==============================================================================

Omnibus: 31.772 Durbin-Watson: 1.960

Prob(Omnibus): 0.000 Jarque-Bera (JB): 16.533

Skew: 0.011 Prob(JB): 0.000257

Kurtosis: 2.449 Cond. No. 294.

==============================================================================

Notes:

[1] Standard Errors assume that the covariance matrix of the errors is correctly specified.

OLS Regression Results

==============================================================================

Dep. Variable: T_PARANOIDSCALE_2 R-squared: 0.140

Model: OLS Adj. R-squared: 0.138

Method: Least Squares F-statistic: 72.18

Date: Tue, 18 Mar 2025 Prob (F-statistic): 3.01e-43

Time: 22:49:50 Log-Likelihood: -5046.8

No. Observations: 1333 AIC: 1.010e+04

Df Residuals: 1329 BIC: 1.012e+04

Df Model: 3

Covariance Type: nonrobust

================================================================================================

coef std err t P>|t| [0.025 0.975]

------------------------------------------------------------------------------------------------

Intercept 49.2074 0.910 54.056 0.000 47.422 50.993

q0002[T.Male] -0.8825 2.075 -0.425 0.671 -4.953 3.188

ACEIQBINARY_v2 1.8846 0.142 13.248 0.000 1.606 2.164

ACEIQBINARY_v2:q0002[T.Male] -0.1540 0.327 -0.471 0.638 -0.795 0.487

==============================================================================

Omnibus: 27.606 Durbin-Watson: 1.839

Prob(Omnibus): 0.000 Jarque-Bera (JB): 23.139

Skew: -0.252 Prob(JB): 9.45e-06

Kurtosis: 2.597 Cond. No. 48.5

==============================================================================

Notes:

[1] Standard Errors assume that the covariance matrix of the errors is correctly specified.

OLS Regression Results

==============================================================================

Dep. Variable: T_PARANOIDSCALE_2 R-squared: 0.183

Model: OLS Adj. R-squared: 0.178

Method: Least Squares F-statistic: 40.70

Date: Tue, 18 Mar 2025 Prob (F-statistic): 6.51e-52

Time: 22:49:51 Log-Likelihood: -4824.7

No. Observations: 1284 AIC: 9665.

Df Residuals: 1276 BIC: 9707.

Df Model: 7

Covariance Type: nonrobust

=====================================================================================================

coef std err t P>|t| [0.025 0.975]

-----------------------------------------------------------------------------------------------------

Intercept 50.8283 0.956 53.156 0.000 48.952 52.704

age_group[T.30-44] -10.9555 2.889 -3.793 0.000 -16.622 -5.289

age_group[T.45-59] -3.2164 2.508 -1.283 0.200 -8.137 1.704

age_group[T.60-81] -2.1867 4.302 -0.508 0.611 -10.626 6.253

ACEIQBINARY_v2 1.7782 0.151 11.783 0.000 1.482 2.074

ACEIQBINARY_v2:age_group[T.30-44] 0.8728 0.401 2.175 0.030 0.085 1.660

ACEIQBINARY_v2:age_group[T.45-59] -0.6692 0.415 -1.614 0.107 -1.483 0.144

ACEIQBINARY_v2:age_group[T.60-81] -1.0381 0.819 -1.267 0.205 -2.645 0.569

==============================================================================

Omnibus: 16.388 Durbin-Watson: 2.023

Prob(Omnibus): 0.000 Jarque-Bera (JB): 15.710

Skew: -0.237 Prob(JB): 0.000388

Kurtosis: 2.736 Cond. No. 99.1

==============================================================================

Notes:

[1] Standard Errors assume that the covariance matrix of the errors is correctly specified.

OLS Regression Results

==============================================================================

Dep. Variable: T_PARANOIDSCALE_2 R-squared: 0.169

Model: OLS Adj. R-squared: 0.166

Method: Least Squares F-statistic: 53.91

Date: Tue, 18 Mar 2025 Prob (F-statistic): 4.82e-51

Time: 22:49:51 Log-Likelihood: -5009.0

No. Observations: 1330 AIC: 1.003e+04

Df Residuals: 1324 BIC: 1.006e+04

Df Model: 5

Covariance Type: nonrobust

===============================================================================================================

coef std err t P>|t| [0.025 0.975]

---------------------------------------------------------------------------------------------------------------

Intercept 54.9194 4.035 13.611 0.000 47.004 62.835

educ[T.Post-graduate degree] -9.0131 4.255 -2.118 0.034 -17.360 -0.667

educ[T.University] -4.4154 4.168 -1.059 0.290 -12.593 3.762

ACEIQBINARY_v2 1.0523 0.532 1.979 0.048 0.009 2.096

ACEIQBINARY_v2:educ[T.Post-graduate degree] 0.7760 0.574 1.352 0.177 -0.350 1.902

ACEIQBINARY_v2:educ[T.University] 0.8002 0.557 1.437 0.151 -0.292 1.893

==============================================================================

Omnibus: 19.660 Durbin-Watson: 1.935

Prob(Omnibus): 0.000 Jarque-Bera (JB): 18.004

Skew: -0.237 Prob(JB): 0.000123

Kurtosis: 2.685 Cond. No. 196.

==============================================================================

Notes:

[1] Standard Errors assume that the covariance matrix of the errors is correctly specified.

OLS Regression Results

==============================================================================

Dep. Variable: T_PARANOIDSCALE_2 R-squared: 0.186

Model: OLS Adj. R-squared: 0.183

Method: Least Squares F-statistic: 59.56

Date: Tue, 18 Mar 2025 Prob (F-statistic): 6.09e-56

Time: 22:49:51 Log-Likelihood: -4903.5

No. Observations: 1305 AIC: 9819.

Df Residuals: 1299 BIC: 9850.

Df Model: 5

Covariance Type: nonrobust

=====================================================================================================================

coef std err t P>|t| [0.025 0.975]

---------------------------------------------------------------------------------------------------------------------

Intercept 53.2814 5.721 9.313 0.000 42.057 64.506

relationship[T.Married/Common-law] -8.7601 6.018 -1.456 0.146 -20.567 3.047

relationship[T.Single] -3.4845 5.792 -0.602 0.548 -14.847 7.878

ACEIQBINARY_v2 0.6701 0.765 0.876 0.381 -0.830 2.171

ACEIQBINARY_v2:relationship[T.Married/Common-law] 1.0502 0.819 1.283 0.200 -0.556 2.656

ACEIQBINARY_v2:relationship[T.Single] 1.2826 0.778 1.649 0.099 -0.244 2.809

==============================================================================

Omnibus: 25.234 Durbin-Watson: 1.953

Prob(Omnibus): 0.000 Jarque-Bera (JB): 22.342

Skew: -0.263 Prob(JB): 1.41e-05

Kurtosis: 2.634 Cond. No. 294.

==============================================================================

Notes:

[1] Standard Errors assume that the covariance matrix of the errors is correctly specified.
